# The Deletion of Endothelial Sodium Channel α (αENaC) Impairs Endothelium-Dependent Vasodilation and Endothelial Barrier Integrity in Endotoxemia *in Vivo*

**DOI:** 10.3389/fphar.2018.00178

**Published:** 2018-04-10

**Authors:** Magdalena Sternak, Anna Bar, Mateusz G. Adamski, Tasnim Mohaissen, Brygida Marczyk, Anna Kieronska, Marta Stojak, Kamil Kus, Antoine Tarjus, Frederic Jaisser, Stefan Chlopicki

**Affiliations:** ^1^Jagiellonian Centre for Experimental Therapeutics, Jagiellonian University, Kraków, Poland; ^2^Chair of Pharmacology, Jagiellonian University Medical College, Kraków, Poland; ^3^Chair and Department of Pharmacy, Jagiellonian University Medical College, Kraków, Poland; ^4^INSERM UMRS1138, Centre de Recherche des Cordeliers, Université Pierre et Marie Curie, Paris, France; ^5^INSERM, Clinical Investigation Centre 1433, Vandœuvre-lès-Nancy, France

**Keywords:** αENaC, endothelium, LPS, endothelial-induced vasodilation, endothelial barrier integrity

## Abstract

The role of epithelial sodium channel (ENaC) activity in the regulation of endothelial function is not clear. Here, we analyze the role of ENaC in the regulation of endothelium-dependent vasodilation and endothelial permeability *in vivo* in mice with conditional αENaC subunit gene inactivation in the endothelium (endo-αENaC^KO^ mice) using unique MRI-based analysis of acetylcholine-, flow-mediated dilation and vascular permeability. Mice were challenged or not with lipopolysaccharide (LPS, from *Salmonella typhosa*, 10 mg/kg, i.p.). In addition, changes in vascular permeability in *ex vivo* organs were analyzed by Evans Blue assay, while changes in vascular permeability in perfused mesenteric artery were determined by a FITC-dextran-based assay. In basal conditions, Ach-induced response was completely lost, flow-induced vasodilation was inhibited approximately by half but endothelial permeability was not changed in endo-αENaC^KO^ vs. control mice. In LPS-treated mice, both Ach- and flow-induced vasodilation was more severely impaired in endo-αENaC^KO^ vs. control mice. There was also a dramatic increase in permeability in lungs, brain and isolated vessels as evidenced by *in vivo* and *ex vivo* analysis in endotoxemic endo-αENaC^KO^ vs. control mice. The impaired endothelial function in endotoxemia in endo-αENaC^KO^ was associated with a decrease of lectin and CD31 endothelial staining in the lung as compared with control mice. In conclusion, the activity of endothelial ENaC *in vivo* contributes to endothelial-dependent vasodilation in the physiological conditions and the preservation of endothelial barrier integrity in endotoxemia.

## Introduction

The epithelial sodium channel (ENaC), composed of three subunits (αENaC, βENaC, and γENaC), is a member of the ENaC/degenerin superfamily of cation-selective ion channels (Canessa et al., 1994; Kosari et al., 1998; Alvarez De La Rosa et al., 2000; Warnock et al., 2014). ENaC is present in the renal epithelium, where it plays an important role in the regulation of renal sodium homeostasis. The expression of ENaC has also been described in the vascular smooth muscle and endothelium, pointing to its possible role in the regulation of vascular function. Indeed, expression of ENaC was reported in cultured human endothelial cells, such as HMEC (Pérez et al., 2009; Wang et al., 2009; Guo et al., 2016), the ECV 304 cell line (Golestaneh et al., 2001) and HUVEC (Kusche-Vihrog et al., 2008) as well as in the endothelium of intact vessels (Pérez et al., 2009; Liu et al., 2015). All three subunits of ENaC are present in endothelium (Pérez et al., 2009), and the αENaC was shown to be involved in the regulation of endothelial cortical stiffness (Jeggle et al., 2013).

In the last decade, a number of reports have described the functional role of endothelial aENaC not only in the control of endothelial nanomechanics, but also vascular resistance and development of endothelial dysfunction. Jeggle et al. (2013) claimed a direct correlation between ENaC surface expression and the formation of cortical stiffness in endothelial cells. The absence of αENaC in endothelial cells led to lower cortical stiffness, while increased αENaC expression induced elevated cortical stiffness. Furthermore, in a mouse model of Liddle syndrome, an inherited form of hypertension caused by gain-of-function mutations in the epithelial Na(+) channel (ENaC), enhanced ENaC expression and increased cortical stiffness were observed in vascular endothelial cells *in situ*, suggesting that ENaC in the vascular endothelium determines endothelial mechanics and vascular function.

The ENaC expression in endothelium is regulated by aldosterone, as in the renal collecting duct cells (Kusche-Vihrog et al., 2010). The aENaC subunit is involved in aldosterone-modulated endothelial stiffness (Jeggle et al., 2013). Aldosterone also increased the amount of ENaC, while blocking the ENaC or aldosterone by amiloride and spironolactone, respectively, led to the disappearance of ENaC channel expression from the cell surface and intracellular pools, reducing cellular content of ENaC protein (Kusche-Vihrog et al., 2008). It was postulated that the inhibition of ENaC channels increased NO production and flow-mediated vasodilation and contributed to improved nanomechanical properties of endothelium (Kusche-Vihrog et al., 2010). In turn, aldosterone may promote endothelial dysfunction by modulating ENaC expression and activity (Pérez et al., 2009). Aldosterone-dependent activation of ENaC in endothelial cells was proposed to be responsible for high salt-induced loss of vasorelaxation in Dahl salt-sensitive (SS) rats (Wang et al., 2017). Not only aldosterone, but also Ox-LDL (oxidized low-density lipoprotein) has been found to stimulate ENaC activity in endothelial cells. This mechanism involves LOX-1 receptor (lectin-like ox-LDL receptor-1)-mediated activation of NADPH oxidase (nicotinamide adenine dinucleotide phosphate-oxidase) and the inhibition of ENaC protects the endothelium from ox-LDL-induced dysfunction (Liang et al., 2017). Interestingly, ENaCs are sensitive to stretch pressure and shear stress and responded to shear stress by increasing the Na (+) influx that could also contribute to endothelium dysfunction (Guo et al., 2016). On the other hand, the work of Liu et al. (2015) showed that reduced ENaC activity was associated with augmented endothelium-dependent relaxation in mesenteric artery in Sprague-Dawley rats challenged with high-salt.

In Na^+^-transporting epithelia, the α subunit of the ENaC is crucial for promoting Na^+^ reabsorption. In a recent study, the endothelial cell barrier protective effect of ENaC-α was demonstrated in pulmonary microvasculature (Czikora et al., 2017). TNF-derived TIP peptide, directly binding to ENaC-α increased both expression and open probability of ENaC in the presence of pore-forming toxin, PLY, which is a major virulence factor and a cause of acute lung injury in *Streptococcus pneumoniae* infection.

Altogether, the activity of ENaC was suggested to contribute to endothelial stiffness, impaired NO production and aldosterone-induced endothelial dysfunction in the endothelium of conduit vessels, but also in the regulation of the integrity of the capillary barrier in the microvascular endothelium. Some discrepancies about the role of ENaC in the regulation of endothelial function may have been related to the heterogeneity of endothelium in macro and microvasculature (Pérez et al., 2009; Jeggle et al., 2013; Guo et al., 2016; Czikora et al., 2017). However, it is important to emphasize that, to our knowledge, none of previous experimental studies analyzing the role of ENaC in the regulation of endothelial function were performed with *in vivo* measurements, but only in *ex vivo* vascular preparations or *in vitro* experiments in cultured endothelial cells. Furthermore, pharmacological tools (such as amiloride or benzamil) were often used to inhibit ENaC, and these drugs may be non-specific for ENaC, particularly at high concentrations (Jia et al., 2016). Genetic deletion of ENaC was used only in some reports (Jeggle et al., 2013; Czikora et al., 2017) and only recently, endo-αENaC^KO^ mice have been generated (Tarjus et al., 2017).

In the present study, we analyzed the role of endothelial αENaC in the regulation of endothelial-dependent vasodilation and vascular permeability in an *in vivo* setting using a unique MRI-based analysis of endothelial function *in vivo* (Bar et al., 2016). Ach-, flow-induced dilation and vascular permeability were assessed in a murine model with targeted inactivation of αENaC in the endothelium (endo-αENaC^KO^ mice) (Tarjus et al., 2017) and control littermates that were challenged or not with LPS (from *Salmonella enterica* serotype abortus equi, 10 mg/kg, i.p.). In addition, changes in vascular permeability were analyzed by EB assay *ex vivo*, while changes in vascular permeability were determined by a FITC-dextran-based assay in isolated, cannulated, and perfused mesenteric artery.

## Materials and Methods

### Animals and Protocol

Endo-αENaC^KO^ mice and their control littermates were generated at the Cordelier Research Centre in Paris, France as recently described (Tarjus et al., 2017). αENaC knockout mice were obtained crossing αENaCf/f floxed mice, kindly provided by Bernard Rossier in Lausanne (Switzerland) with transgenic mice expressing Cre recombinase under the control of Tie2 promoter on a C57Bl/6 genetic background (The Jackson Laboratory, United States). αENaCf/f littermates lacking the Tie2-Cre transgene were used as controls.

Endo-αENaC^KO^ and control mice were kept under controlled conditions (22–24°C, 55% humidity, 12 h day/night rhythm with free access to food and water until the day of experiment). The animal procedures described in the present study were approved by the local Jagiellonian University Ethical Committee on Animal Experiments, in accordance with the Guidelines for Animal Care and Treatment of the European Community.

To induce endotoxemia, LPS (from *Salmonella typhosa*, Sigma–Aldrich, St. Louis, MO, United States) was injected intraperitoneally (10 mg/kg).

The endothelial function, permeability changes and other final surgical procedures (collection of blood and tissues for Western Blot or histological examinations) were taken 12 h after LPS administration after anesthetization of mice with ketamine and xylazine (100 mg/kg and 10 mg/kg, respectively, i.p. Pfizer, New York, NY, United States). Control animals were always treated with intraperitoneal injections of adequate volumes of saline.

### MRI Protocol for the Assessment of Endothelial Function *in Vivo*

MRI experiments were performed using a 9.4T scanner (BioSpec 94/20 USR, Bruker, BioSpin GmbH, Germany), as described previously (Bar et al., 2016). Mice were anesthetized using isoflurane (Aerrane, Baxter Sp. z o. o., Warszawa, Poland, 1.7 vol. %) in an oxygen and air (1:2) mixture. Body temperature was maintained at 37°C using circulating warm water. ECG, respiration and body temperature were monitored using a Model 1025 Monitoring and Gating System (SA Inc., Stony Brook, NY, United States).

Endothelium-dependent vascular responses *in vivo* were assessed using two techniques: endothelium-dependent response to Ach administration, as described previously (Bar et al., 2015, 2016) and FMD in response to reactive hyperemia, considered as a gold standard for clinical studies of endothelial dysfunction (Raitakari and Celermajer, 2000; Frolow et al., 2015). Response to injection of Ach (Sigma–Aldrich, Poznañ, Poland: 50 μl, 16.6 mg/kg, i.p.), was analyzed in the lower part of the TA, whereas FMD after short-term occlusion (home-made vessel occluder – description in Supplementary Materials) was determined in the FA. Vasomotor response was examined by comparing two, time-resolved 3D images of the vessels prior to and 25 min after intraperitoneal Ach administration (time was determined experimentally in our previous study (Bar et al., 2016) or after vessel occlusion had lasted 5 min. The plasma level of Ach achieved with intraperitoneally injection was determined using LC/MS/MS technique based on previous report (Kirsch et al., 2010), with minor modifications The plasma concentration of Ach was increased already after 10 min and maintained elevated 25 min after administration as compared to the control: (0.07 ± 0.13 nM; 2.08 ± 1.97 nM, 0.58 ± 0.78 nM, before, 10 and 25 min after Ach, respectively). Images were acquired using the cine IntraGate^TM^ FLASH 3D sequence, reconstructed with the IntraGate 1.2.b.2 macro (Bruker). End-diastolic volumes of vessels were analyzed using ImageJ software 1.46r (NIH, Bethesda, MD, United States) and scripts written in Matlab (MathWorks, Natick, MA, United States). Imaging parameters included the following: repetition time (TR) – 6.4 ms, echo time (TE) – 1.4 ms, field of view (FOV) – 30 mm × 30 mm × 5 mm, matrix size – 256 × 256 × 30, flip angle (FA) – 30°, and number of accumulations (NA) – 15, reconstructed to seven cardiac frames. Total scan time was 10 min.

Data analysis: 3D images of TA were positioned on sagittal view of the mice, about 5 mm under the heart. 3D images of FA were positioned on the coronal view of the mice, on the left hind limb of the mouse. All cross-sectional areas of vessels at each slice were obtained using thresholding segmentation in ImageJ and exported to Matlab, where vessel volumes were reconstructed and calculated.

### MRI – *In Vivo* Assessment of Vascular Permeability

Mice were imaged in the supine position to assess endothelial permeability. Relaxation time (T_1_) maps were measured using the cine IntraGate^TM^ FLASH 3D sequence and the variable flip angle (VFA) technique. Obtained T_1_ maps, before and 30 mins after intravenous administration of albumin-binding gadolinium contrast agent (CA: Galbumin, BioPal, Worcester, MA – 25 mg/ml, 4.5 ml/kg) were compared pixel by pixel, using scripts written in Matlab (MathWorks, Natick, MA, United States). As a result of this comparison, the number of pixels for which T_1_ had changed more than 50% after contrast agent administration (Npx50) was calculated. Npx50 was proposed as an alternative method for the assignment of the changes in the endothelial permeability. Indeed, the size of the region of interest (ROI) was difficult to establish objectively, and the idea of finding the pixels for which T1 had changed by more than 50% allows for operator-independent assessment of the ROI around the vessel (Bar et al., 2016), NMR in biomed. Increased value of the Npx50 is associated with increased endothelial permeability. Imaging parameters for endothelial permeability assessment included the following: TR – 10 ms, TE – 1.1 ms, FOV – 30 mm × 30 mm × 4 mm, matrix size – 192 × 160 × 8, number of repetitions – 12, and reconstructed to one cardiac frame. Eight FA were used: 2°, 4°, 6°, 8°, 14°, 20°, 30°, and 50°. FA values were set by changing the length of a radiofrequency pulse, with constant amplifier power. Total scan time for all angles was 16 min.

### BBB – *In Vivo* Assessment of Vascular Permeability

Subsequent to anesthesia (100 mg/kg ketamine + 10 mg/kg xylazine, i.p.), mice were injected (femoral vein) with a solution of EB (Sigma–Aldrich) at a dose of 4 ml/kg. Dye solution comprised 2% EB in 0.9% saline. Dyes were left to circulate for 10 min, then the mice chest was surgically opened to simultaneously perfuse left (systemic circulation) and right ventricle (pulmonary circulation) with PBS for 15 min. Lungs and brain were isolated and brain was separated to cerebral cortex, hippocampus, cerebellum and brainstem. Isolated brain structures and lungs were dry-weighted and homogenized in 200 μl of 50% TCA (dissolved in distilled water). Homogenate was frozen and kept at -20°C for dye concentration measurement. Subsequent to thawing, homogenates were centrifuged (at 12,000 rpm for 12 min at 4°C) and the supernatant was collected and diluted with 1:3 volumes of 95% ethanol prior to photospectrometric (Synergy 4, Bio-Tek) determination of EB concentration (fluorescence: excitation at 590 nm, emission at 645 nm; absorbance at 620 nm). Results were normalized to the tissue weight.

### *Ex Vivo* Assessment of Vascular Permeability

In order to assess vascular permeability *ex vivo*, the first branch mesenteric artery was gently isolated and freed from adhering tissue in PSS NaCl 130 mM; NaHCO_3_ 15 mM; KCl 3.7 mM; NaHCO_3_ 15 mM; MgSO_4_ 1.2 mM; glucose 11 mM; CaCl_2_ 1.6 mM and HEPES 5 mM) under a dissecting microscope. Then, the mesenteric artery was mounted on a pressure myograph (DMT, Danish Myo Technology A/S, Aarhus, Denmark), pressurized under no flow at 60 mmHg and incubated for an additional half an hour at 37°C. The PSS solution was continuously aerated with gas (containing 74% N_2_, 5% CO_2_, and 21% O_2_), resulting in a pH of 7.4. Diameters were recorded simultaneously with light emitted by arteries at 510 nm with an excitation wavelength of 340 and 380 nm (Ionoptix Corporation, Westwood, MA, United States).

Subsequently, the artery was perfused with dextran-binding fluorescein isothiocyanate (FITC-dextran 150 kDa, Sigma–Aldrich: 50 μg/ml) for 90 mins under flow conditions close to the physiological state (flow ∼ 150–160 μl/min). Fluid from the chamber in which the vessel was submerged was sampled every 15 min for 90 min. The FITC-dextran concentration was assessed in the samples using fluorescence intensity measurements.

### Immunofluorescence and Immunohistochemical Determination of Lectin and CD31 in the Lungs

After anesthesia (100 mg/kg ketamine + 10 mg/kg xylazine, i.p.), small fragments of lung tissue were collected, washed in PBS solution, and then placed in 4% buffered formalin. Tissues were then rinsed, embedded in paraffin in 5 μm sections and placed on poly-L-lysine-covered microscopic slides (Metzel Glaser Super Frost). For immunofluorescence staining, collected slides were stained using lectin (Vector Laboratories) followed by Cy3-conjugated streptavidin (Jackson Immuno Research). Subsequently, 10 randomly chosen eyefields near the regions of microcirculation were photographed for each mouse and subjected to acquisition using an AxioCam MRc5 digital camera and an AxioObserver D1 inverted fluorescent microscope (Zeiss), stored as tiff files and analyzed automatically using Columbus software (version 2.4.2, Perkin Elmer). The automatic thresholding of microscope image was used to extract the signal area from the background. Then, the fluorescent signal was calculated from the extracted area. The results were presented as the relative lectin I immunopositive area to the all tissue area.

For CD31 immunohistochemical staining, collected slides were stained with rabbit anti-mouse CD31 (Abcam), followed by goat-anti-rabbit secondary antibody (Jackson Immuno Research).

Subsequently, the total area of lung was scanned with a BX51 microscope equipped with the virtual microscopy system dotSlide (Olympus, Japan) and subjected to segmentation in Ilastik software to assess the relative CD31 immunopositive area to the all-tissue area. Image segmentation was performed in Ilastik (developed by the Ilastik team, with partial financial support by the Heidelberg Collaboratory for Image Processing, HHMI Janelia Farm Research Campus and CellNetworks Excellence Cluster) and calculated by Image J program.

### Statistical Analysis

All of the data obtained are presented as mean and standard error of the mean (SEM) or in case of the lack of normal distribution as median with interquartile range. Statistical tests were done using GraphPad Prism 5 (GraphPad Software, Inc., La Jolla, CA, United States) software. Non-parametric test (Kruskal–Wallis test followed by Dunn’s *post hoc* test) or parametric test (one-way ANOVA followed by Tukey’s *post hoc* test) were performed. Statistical significance was defined as *p* < 0.05.

## Results

### Acetylcholine- and Flow-Induced Vasodilatation in Endo-αENaC^KO^ and Control Mice in Basal Conditions and in Endotoxemia Assessed *in Vivo* by MRI

**FIGURE 1:**
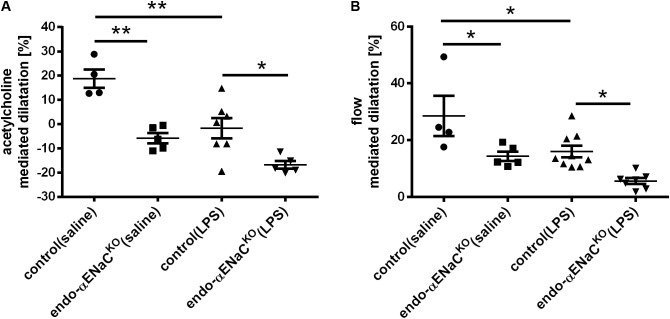
**(A)** Acetylcholine- (Ach, 16.6 mg/kg, dissolved in 50 μl pyrogen-free saline administered intraperitoneally) and **(B)** flow-dependent dilation in endo-αENaC^KO^ and control mice in basal conditions (saline) and in endotoxemia (LPS, 10 mg/kg, i.p., 12 h) assessed *in vivo* by MRI. Ach-dependent dilation was measured in TA while flow-dependent dilation was determined in FA, control (saline) *n* = 4, endo-αENaC^KO^ (saline) *n* = 5, control (LPS) *n* = 7–9, endo-αENaC^KO^ (LPS) *n* = 5–7. Statistics: one-way ANOVA followed by Tukey’s *post hoc* test (normality was assessed using the Kolmogorov–Smirnov test). The results are presented as the mean ± SEM, ^∗^*p* < 0.05, ^∗∗^*p* < 0.01.

Intraperitoneal injection of Ach in control mice resulted in vasodilatation with the peak response occurring 25 min following Ach administration. In endo-αENaC^KO^ mice, Ach-induced response was totally lost, while flow-induced vasodilation was inhibited approximately by half as compared with control mice. In the endotoxemia setting (i.e., after LPS injection), both Ach- and flow-induced vasodilation were more severely impaired in endo-αENaC^KO^ mice as compared with control mice (**Figures 1A,B**).

### Endothelial Permeability in Endo-αENaC^KO^ and Control Mice in Basal Conditions and in Endotoxemia Assessed *in Vivo* by MRI and T_1_ Mapping of Gd-Albumin Contrast Agent Accumulation in the Vessel Wall

**FIGURE 2:**
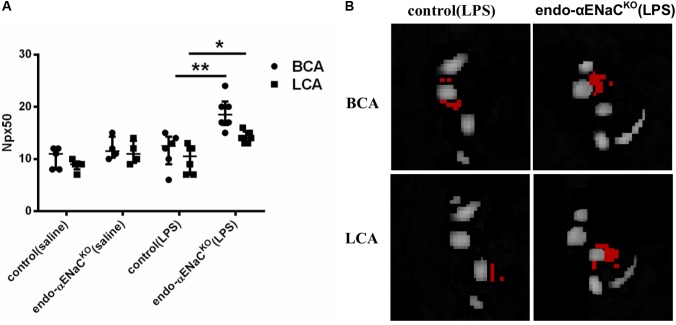
**(A)** Changes in number of pixels (Npx50) for which T1 had changed around BCA and LCA by about 50%, 30 min after Galbumin contrast agent administration in endo-αENaC^KO^ and control mice in basal conditions (saline) and in endotoxemia (10 mg/kg, i.p., 12 h) assessed *in vivo* by MRI. **(B)** Representative images of BCA and LCA cross sections, in which Npx50 is marked in red, control (saline) *n* = 5, endo-αENaC^KO^ (saline) *n* = 4, control (LPS) *n* = 6, endo-αENaC^KO^ (LPS) *n* = 6. Statistics: for BCA: one-way ANOVA followed by Tukey’s *post hoc* test; for LCA: Kruskal–Wallis test followed by Dunn’s *post hoc* test (normality was assessed using the Kolmogorov–Smirnov test). The results are presented as the median with interquartile range, ^∗^*p* < 0.05, ^∗∗^*p* < 0.01.

In basal conditions, the endothelial barrier integrity was preserved both in endo-αENaC^KO^ and control mice. However, in endotoxemia, permeability was increased in endo-αENaC^KO^ mice as compared with control mice 12 h after LPS injection, as evidenced by an increased Npx50 parameter as defined in our previous work (Bar et al., 2016). Permeability of the endothelium differed in endo-αENaC^KO^ mice as compared with controls along the whole length of the BCA and LCA (**Figures 2A,B**).

### Endothelial Permeability in the Perfused Lungs, Liver, and Blood–Brain Barrier (BBB) in Endo-αENaC^KO^ and Control Mice in Basal Conditions and in Endotoxemia Assessed *ex Vivo* by Evans Blue

**FIGURE 3:**
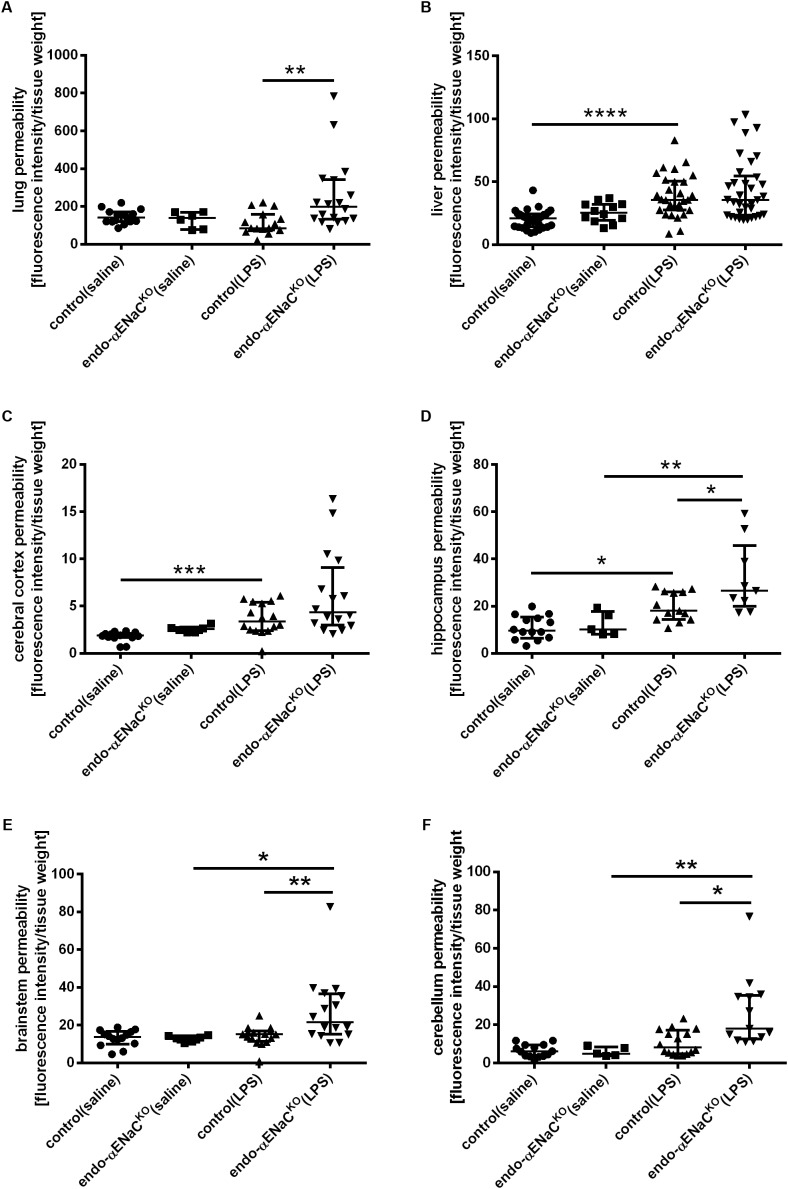
Changes in fluorescence intensity in homogenates of perfused organs: **(A)** lungs, **(B)** liver **(C–F)** blood–brain barrier (BBB) in endo-αENaC^KO^ and control mice in basal conditions (saline) and in endotoxemia (10 mg/kg, i.p., 12 h). EB was administered as the contrast agent (4 mg/ml, i.v.), control (saline) *n* = 14–32, endo-αENaC^KO^ (saline) *n* = 5–12, control (LPS) *n* = 13–30, endo-αENaC^KO^ (LPS) *n* = 9–36. Statistics: for hippocampus, brainstem: one-way ANOVA followed by Tukey’s *post hoc* test; for lung, liver, cerebral cortex, cerebellum: Kruskal–Wallis test followed by Dunn’s *post hoc* test (normality was assessed using the Kolmogorov–Smirnov test). The results are presented as the median with interquartile range, ^∗^*p* < 0.05, ^∗∗^*p* < 0.01, ^∗∗∗^*p* < 0.001; ^∗∗∗^*p* < 0.0001.

As shown in **Figures 3A–F**, the barrier integrity was not changed in the microcirculation of lungs, liver and brain in both endo-αENaC^KO^ and control mice in basal conditions. On the contrary, in endotoxemia, BBB with the exception of cerebral cortex, was significantly impaired in the hippocampus, brainstem and cerebellum of endo-αENaC^KO^ mice as compared with control mice. After LPS, the lung permeability was also significantly increased in endo-αENaC^KO^ mice as compared with control mice. Of note, the barrier integrity of liver was not changed both in endo-αENaC^KO^ and control mice after LPS administration.

### Endothelial Permeability in the Isolated, Cannulated Vascular Preparation in Endo-αENaC^KO^ and Control Mice in Basal Conditions and in Endotoxemia Assessed *ex Vivo* by FITC-dextran

**FIGURE 4:**
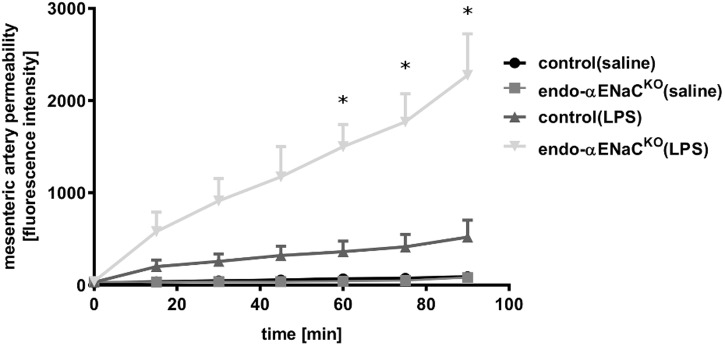
Changes in fluorescence intensity in PSS buffer measured outside the cannulated vessel along 90 min of the perfusion from endo-αENaC^KO^ and control mice in basal conditions and in endotoxemia (10 mg/kg, i.p., 12 h). FITC-dextran (150 kDa, 50 μg/ml) was administered as the contrast agent, control (saline) *n* = 7, endo-αENaC^KO^ (saline) *n* = 6, control (LPS) *n* = 7, endo-αENaC^KO^ (LPS) *n* = 6. Statistics: Kruskal–Wallis test followed by Dunn’s *post hoc* test (normality was assessed using the Kolmogorov–Smirnov test). The results are presented as the median with interquartile range, ^∗^*p* < 0.05.

In basal conditions, the permeability of perfused mesenteric artery was not changed both in endo-αENaC^KO^ and control mice. In contrast, in endotoxemia, permeability of perfused artery in endo-αENaC^KO^ mice was increased as compared with controls. The increased permeability, measured as the change in extravascular FITC-dextran concentration in the vessel incubation chamber, increased significantly during 90 min of perfusion (**Figure 4**).

### Expression of Lectin I, CD31, in the Lung of Endo-αENaC^KO^ and Control Mice in Basal Conditions and in Endotoxemia

**FIGURE 5:**
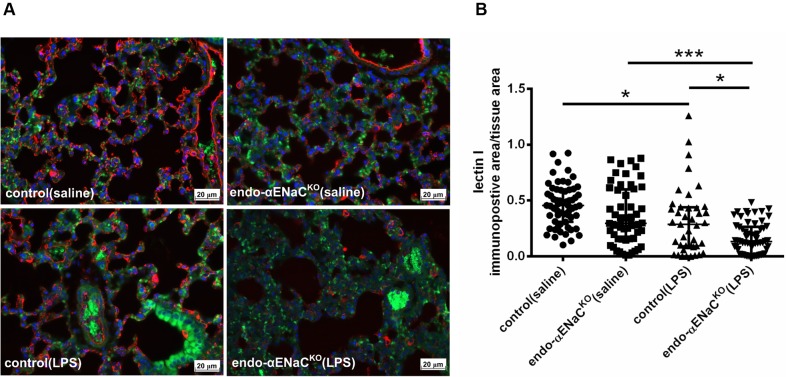
**(A)** Immunofluorescent images of lung microcirculation stained with glycocalyx marker – lectin I (labeled by Cy3, red channel) in endo-αENaC^KO^ and control mice in basal conditions (saline) and in endotoxemia. **(B)** Quantification of immunofluorescent staining for lectin I. Ten randomly chosen eyefields near the regions of microcirculation were photographed for each mouse and subjected to quantification of relative immunopositive area to the all tissue area with the Columbus software, control (saline) *n* = 7, endo-αENaC^KO^ (saline) *n* = 7, control (LPS) *n* = 8, endo-αENaC^KO^ (LPS) *n* = 8. Statistics: Kruskal–Wallis test followed by Dunn’s *post hoc* test (normality was assessed using the Kolmogorov–Smirnov test). The results are presented as the median with interquartile range, ^∗^*p* < 0.05, ^∗∗∗^*p* < 0.001

**FIGURE 6:**
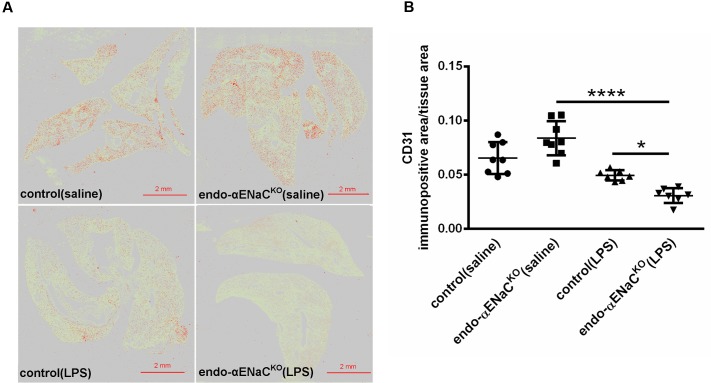
**(A)** Immunohistochemical images of lung stained with endothelial marker - CD31 after Ilastik segmentation (immunopositive area marked in red) in endo-αENaC^KO^ and control mice in basal conditions (saline) and in endotoxemia. **(B)** Quantification of immunohistochemical staining for CD31. Immunohistochemical signal was quantified with the Ilastik program to assess the relative CD31 immunopositive area to the all-tissue area after scanning all images with BX51 microscope equipped with virtual microscopy system dotSlide (Olympus, Japan), control (saline) *n* = 8, endo-αENaC^KO^ (saline) *n* = 8, control (LPS) *n* = 7, endo-αENaC^KO^ (LPS) *n* = 7. Statistics: one-way ANOVA followed by Tukey’s *post hoc* test (normality was assessed using the Kolmogorov–Smirnov test). The results are presented as the mean ± SEM, ^∗^*p* < 0.05, ^∗∗^*p* < 0.01, ^∗∗∗∗^*p* < 0.0001.

In basal conditions, the immunofluorescent (IF) and immunohistochemical (IHC) staining of lung microcirculation did not show significant changes in expression of lectin I (IF) and CD31 (IHC) both in endo-αENaC^KO^ and control mice. In contrast, in endotoxemia, the down-regulation of lectin I and CD31 was observed in lungs of endo-αENaC^KO^ as compared with control mice (**Figures 5**, **6**).

## Discussion

Using a cell-specific knockout mouse model with deletion of the αENaC subunit in the endothelial cells, we demonstrated *in vivo* that genetic deletion of the αENaC subunit in the endothelium resulted in blunted Ach- and flow-induced vasodilation in the aorta and FA, respectively, without a major effect on endothelial permeability. In endotoxemia (induced by LPS, 10 mg/kg), the absence of endothelial ENaC resulted in a more severe impairment of Ach- and flow-induced vasodilation in conduit vessels, as well as pronounced endothelial barrier dysfunction in conduit and peripheral vessel as well as in the lung and brain microcirculation in comparison with mice with preserved endothelial ENaC expression. This dysregulation of the endothelial barrier was associated with altered glycocalyx in endo-αENaC^KO^ mice as evidenced by the lower expression of lectin and CD31 in lungs Altogether, our comprehensive study using various methods to detect endothelial function and permeability changes in various vascular beds allows us to suggest that endothelial ENaC contributes to endothelial-dependent regulation of vascular tone in conduit vessels and to the preservation of the endothelial barrier function in endotoxemia both in conduit vessels, in the peripheral circulation as well as in the microcirculation of the lung and brain.

The use of a cell-specific knockout mouse model – endo-αENaC^KO^ mice – allowed us to describe for the first time the involvement of endothelial αENaC in the regulation of endothelial function *in vivo*. In the present work, we used an MRI-based method to measure endothelium-dependent vasodilation and endothelial permeability *in vivo* as described previously (Bar et al., 2016). We assessed endothelial function *in vivo* in the aorta, in response to administration of Ach, and in the FA in response to flow (flow-mediated vasodilation, FMD). MRI-based assessment of endothelial permeability changes was performed for BCA and LCA, where the leakage through intercellular junctions of endothelium was evidenced by accumulation of albumin-binding contrast agent in vessel walls (Lobbes et al., 2009; Pedersen et al., 2011) and analyzed as shortening of the T_1_ in the vessel and Npx50-based operator-independent assessment of endothelial permeability (Bar et al., 2016).

Our results indicating that endothelial deletion of ENaC impaired endothelium-dependent responses are only partially compatible with the work of Tarjus et al. (2017). By using the same cell-specific knockout mouse model, Tarjus et al. (2017) showed that acute treatment with benzamil, a pharmacological antagonist of ENaC, decreased Ach-mediated NO production. However in endo-αENaC^KO^ mice Ach-induced NO release was preserved while flow-mediated dilation was impaired. Results of Tarjus et al. (2017) are not in line with our results as regards Ach-induced vasodilation but concordant with our results as regards flow-induced vasodilation. Heterogeneity of endothelium in macro and microvasculature may explain the apparent discrepancy between our result (showing impaired agonist and flow-mediated dilation in endo-αENaC^KO^ mice) and previous results from Tarjus et al. (2017) (showing only the impairment in flow-mediated dilation). Our study was performed using *in vivo* studies in conduit vessels, while those of Tarjus et al. (2017) were done in resistance mesenteric arteries. Given the facts that contribution of NO to endothelium-dependent vasodilation is greater in larger vessels (Campbell et al., 1996), different effects of endothelial αENaC knock out on Ach-induced vasodilation in conduit and resistance artery may related to various contributions of NO to endothelium-dependent vasodilation induced by Ach. Of note, deletion of αENaC did not affect the basal diameter of the artery. The basal volume of aorta measured by MRI before adding Ach was similar in all four experimental groups of mice.

Our results are in contrast with the studies of Liu et al. (2015) showing increased Ach-mediated vasodilation after amiloride pre-treatment, or Jernigan and Drummond (2005) reporting no effect of benzamil on phenylephrine-induced contraction in mouse interlobar arteries. Jia et al. (2016) and Knoepp et al. (unpublished) demonstrated that amiloride improved flow-induced dilatation, suggesting that ENaC antagonist prevents endothelial dysfunction. However, amiloride and benzamil can affect other targets apart from the endothelial ENaC, which makes these results questionable. In contrast, our study took advantage of endo-αENaC^KO^ mice that represent a more selective approach to inactivate ENaC in the endothelium.

Given the important role of Na^+^/Ca^2+^ exchanger in endothelial NO production and endothelium-dependent relaxation (Schneider et al., 2002; Bondarenko et al., 2017), we propose that impairment of endothelium-dependent vasodilation in the absence of ENaC may be mechanistically linked to the function of Na^+^/Ca^2+^ exchanger. This hypothesis, however, remains to be verified.

It is important to highlight that we have demonstrated for the first time an important role of endothelial αENaC in the regulation of endothelial permeability *in vivo*. Among different features of the endothelial dysfunction, increased endothelial permeability is of special importance in various pathophysiological conditions including endotoxemia (Blann, 2003; Davignon and Ganz, 2004; Bar et al., 2015). Since it is well known that the vascular endothelial barrier function plays the crucial role in the maintenance of homeostasis and the integrity of organs in the body (Wiesinger et al., 2013) it is necessary to understand the regulation of this barrier to prevent organ injury. Our results agree with a recent study performed in cultured microvascular pulmonary endothelial cells which demonstrate a previously unrecognized role for αENaC in supporting capillary barrier function that may apply to the human lung (Czikora et al., 2017).

We consider two possibilities to explain the relation between endothelial permeability and glycocalyx disruption. On one hand, ENaC may be directly involved in the regulation of glycocalyx integrity, while on the other hand glycocalyx injury is the consequence of endothelial barrier injury resulting from the loss of ENaC-dependent regulation of endothelial cell permeability. We provide evidence that in the absence of ENaC, LPS challenge resulted in lower expression of lectin I (a specific marker for glycocalyx) and CD31 in lungs from endo-αENaC^KO^ mice as compared to control mice. Interestingly, platelet endothelial cell adhesion molecule (CD31) has a lectin-like activity toward α2,6-sialic acid critical for homophilic interactions and endothelial viability (Kitazume et al., 2014). Thus, loss of CD31 could contribute to endothelial barrier disruption in endo-αENaC^KO^ mice. Given the fact that tight junction rather than adherens junction determine the endothelial permeability we suspect that αENaC activity may be more linked with the regulation of tight junction paracellular permeability rather than adherens junctions (Fernández-Martín et al., 2012; Hu et al., 2013; Ren et al., 2015). Obviously, several signaling pathways not studied here such as Rho/ROCK, PKCs, MAPK or Rho/Rac activity could be involved in regulation of barrier integrity by αENaC (Birukova et al., 2013; Hu et al., 2013; Mammoto et al., 2013; Han et al., 2016; Radeva and Waschke, 2017).

In summary, even though a number of mechanisms could be involved in the αENaC-dependent regulation of barrier function that have been not fully defined here our results univocally suggest that strategies aiming to activate αENaC may represent a novel approach to improve barrier function in the capillary endothelium, not only during pneumonia as suggested previously (Czikora et al., 2017), but also in endotoxemia.

Numerous reports have shown that increased ENaC activity tends to stiffen the endothelium followed by reduced NO release and vasoconstriction (Jeggle et al., 2013; Kusche-Vihrog et al., 2014). Even though under *in vitro* conditions, endothelial cortical stiffness is inversely correlated with NO production (Kusche-Vihrog et al., 2010; Warnock et al., 2014), the fact that endo-αENaC^KO^ mice display a softer cortical layer of endothelium in *ex vivo* aorta (Tarjus et al., 2017) suggests that cortical endothelial stiffness represents a pathophysiological phenomenon not directly linked to endothelial-dependent vasodilation *in vivo.* On the other hand, given the detrimental role of aldosterone on endothelial function in cardiovascular disease and the beneficial effect of aldosterone in endotoxemia (Fadel et al., 2017), our results may underline the differential role of the aldosterone/ENaC pathway in healthy and disease conditions.

## Conclusion

We have demonstrated in this study that endothelial αENaC plays a crucial role in vascular physiology and pathophysiology. In physiological conditions, endothelial αENaC regulates Ach- and flow-induced vasodilation, while in pathophysiological conditions αENaC contributes to the preservation of the endothelial barrier function. Accordingly, our results suggest that it is not the inhibition – as previously suggested – but the stimulation of endothelial αENaC which may be beneficial for improved endothelial function.

## Author Contributions

Conceived and designed the study: MgS and SC. Performed the study: MgS, AB, MA, TM, BM, AK, AT, MtS, and KK. Analyzed the data: MgS, AB, and MA. Provided the analytical tools: AT and FJ. Drafted the manuscript: MgS and SC. Revised the draft of manuscript: FJ. All authors corrected and approved the final version of the manuscript.

## Conflict of Interest Statement

The authors declare that the research was conducted in the absence of any commercial or financial relationships that could be construed as a potential conflict of interest.

## Abbreviations

αENaC: endothelial sodium channel α
Ach: acetylcholine
BCA: brachiocephalic artery
EB: Evans Blue
FA: femoral artery
FMD: flow-mediated dilatation
LCA: left carotid artery
LOX-1 receptor: lectin-like ox-LDL receptor-1
LPS: lipopolysaccharide
NADPH oxidase: nicotinamide adenine dinucleotide phosphate-oxidase
NCX: Na^+^/Ca^2+^ exchanger
Ox-LDL: oxidized low-density lipoprotein
PLY: pneumolysin
TA: thoracic aorta
TCA: trichloroacetic acid

## References

[B1] Alvarez De La Rosa D. Canessa C. M. Fyfe G. K. Zhang P. (2000). Structure and regulation of amiloride- sensitive sodium channels. *Annu. Rev. Physiol.* 62 573–594. 10.1146/annurev.physiol.62.1.573 10845103

[B2] Bar A. Skorka T. Jasinski K. Chlopicki S. (2015). MRI-based assessment of endothelial function in mice *in vivo*. *Pharmacol. Rep.* 67 765–770. 10.1016/j.pharep.2015.05.007 26321279

[B3] Bar A. Skórka T. Jasiñski K. Sternak M. Bartel Z. Tyrankiewicz U. (2016). Retrospectively gated MRI for *in vivo* assessment of endothelium-dependent vasodilatation and endothelial permeability in murine models of endothelial dysfunction. *NMR Biomed.* 29 1088–1097. 10.1002/nbm.3567 27348596

[B4] Birukova A. A. Tian X. Cokic I. Beckham Y. Gardel M. L. Birukov K. G. (2013). Endothelial barrier disruption and recovery is controlled by substrate stiffness. *Microvasc. Res.* 87 50–57. 10.1016/j.mvr.2012.12.006 23296034 PMC3627818

[B5] Blann A. D. (2003). Assessment of endothelial dysfunction: focus on atherothrombotic disease. *Pathophysiol. Haemost. Thromb.* 33 256–261. 10.1159/000083811 15692226

[B6] Bondarenko A. I. Montecucco F. Panasiuk O. Sagach V. Sidoryak N. Brandt K. J. (2017). GPR55 agonist lysophosphatidylinositol and lysophosphatidylcholine inhibit endothelial cell hyperpolarization via GPR-independent suppression of Na^+^-Ca^2+^ exchanger and endoplasmic reticulum Ca^2+^ refilling. *Vascul. Pharmacol.* 89 39–48. 10.1016/j.vph.2017.01.002 28064014 PMC6520228

[B7] Campbell W. B. Gebremedhin D. Pratt P. F. Harder D. R. (1996). Identification of epoxyeicosatrienoic acids as endothelium-derived hyperpolarizing factors. *Circ. Res.* 78 415–423. 8593700 10.1161/01.res.78.3.415

[B8] Canessa C. M. Schild L. Buell G. Thorens B. Gautschi I. Horisberger J. D. (1994). Amiloride-sensitive epithelial Na^+^ channel is made of three homologous subunits. *Nature* 367 463–467. 10.1038/367463a0 8107805

[B9] Czikora I. Alli A. A. Sridhar S. Matthay M. A. Pillich H. Hudel M. (2017). Epithelial sodium channel-α mediates the protective effect of the TNF-derived TIP peptide in pneumolysin-induced endothelial barrier dysfunction. *Front. Immunol.* 8:842. 10.3389/fimmu.2017.00842 28785264 PMC5519615

[B10] Davignon J. Ganz P. (2004). Role of endothelial dysfunction in atherosclerosis. *Circulation* 109(23 Suppl. 1) III27–III32. 10.1161/01.CIR.0000131515.03336.f8 15198963

[B11] Fadel F. André-Grégoire G. Gravez B. Bauvois B. Bouchet S. Sierra-Ramos C. (2017). Aldosterone and vascular mineralocorticoid receptors in murine endotoxic and human septic shock. *Crit. Care Med.* 45 e954–e962. 10.1097/CCM.0000000000002462 28445239

[B12] Fernández-Martín L. Marcos-Ramiro B. Bigarella C. L. Graupera M. Cain R. J. Reglero-Real N. (2012). Crosstalk between reticular adherens junctions and platelet endothelial cell adhesion molecule-1 regulates endothelial barrier function. *Arterioscler. Thromb. Vasc. Biol.* 32 e90–e102. 10.1161/ATVBAHA.112.252080 22723439

[B13] Frolow M. Drozdz A. Kowalewska A. Nizankowski R. Chlopicki S. (2015). Pharmacological reports comprehensive assessment of vascular health in patients; towards endothelium-guided therapy. *Pharmacol. Rep.* 67 786–792. 10.1016/j.pharep.2015.05.010 26321282

[B14] Golestaneh N. Klein C. Valamanesh F. Suarez G. Agarwal M. K. Mirshahi M. (2001). Mineralocorticoid receptor-mediated signaling regulates the Ion gated sodium channel in vascular endothelial cells and requires an intact cytoskeleton. *Biochem. Biophys. Res. Commun.* 280 1300–1306. 10.1006/bbrc.2001.4275 11162670

[B15] Guo D. Liang S. Wang S. Tang C. Yao B. Wan W. (2016). Role of epithelial Na+ channels in endothelial function. *J. Cell Sci.* 129 290–297. 10.1242/jcs.168831 26621031

[B16] Han J. Weisbrod R. M. Shao D. Watanabe Y. Yin X. Bachschmid M. M. (2016). The redox mechanism for vascular barrier dysfunction associated with metabolic disorders: glutathionylation of rac1 in endothelial cells. *Redox Biol.* 9 306–319. 10.1016/j.redox.2016.09.003 27693992 PMC5045950

[B17] Hu Y. J. Wang Y. D. Tan F. Q. (2013). Regulation of paracellular permeability: factors and mechanisms. *Mol. Biol. Rep.* 40 6123–6142. 10.1007/s11033-013-2724-y 24062072

[B18] Jeggle P. Callies C. Tarjus A. Fassot C. Fels J. Oberleithner H. (2013). Epithelial sodium channel stiffens the vascular endothelium in vitro and in liddle mice. *Hypertension* 61 1053–1059. 10.1161/HYPERTENSIONAHA.111.199455 23460285

[B19] Jernigan N. L. Drummond H. A. (2005). Vascular ENaC proteins are required for renal myogenic constriction. *Am. J. Physiol. Renal Physiol.* 289 F891–F901. 10.1152/ajprenal.00019.2005 15914781

[B20] Jia G. Habibi J. Aroor A. R. Martinez-Lemus L. A. Demarco V. G. Ramirez-Perez F. I. (2016). Endothelial mineralocorticoid receptor mediates diet-induced aortic stiffness in females. *Circ. Res.* 118 935–943. 10.1161/CIRCRESAHA.115.308269 26879229 PMC4798906

[B21] Kirsch S. H. Herrmann W. Rabagny Y. Obeid R. (2010). Quantification of acetylcholine, choline, betaine, and dimethylglycine in human plasma and urine using stable-isotope dilution ultra performance liquid chromatography-tandem mass spectrometry. *J. Chromatogr. B Analyt. Technol. Biomed. Life Sci.* 878 3338–3344. 10.1016/j.jchromb.2010.10.016 21074502

[B22] Kitazume S. Imamaki R. Kurimoto A. Ogawa K. Kato M. Yamaguchi Y. (2014). Interaction of platelet endothelial cell adhesion molecule (PECAM) with 26-sialylated glycan regulates its cell surface residency and anti-apoptotic role. *J. Biol. Chem.* 289 27604–27613. 10.1074/jbc.M114.563585 25135639 PMC4183799

[B23] Kosari F. Sheng S. Li J. Mak D. O. Foskett J. K. Kleyman T. R. (1998). Subunit stoichiometry of the epithelial sodium channel. *J. Biol. Chem.* 273 13469–13474. 10.1074/jbc.273.22.13469 9593680

[B24] Kusche-Vihrog K. Callies C. Fels J. Oberleithner H. (2010). The epithelial sodium channel (ENaC): mediator of the aldosterone response in the vascular endothelium? *Steroids* 75 544–549. 10.1016/j.steroids.2009.09.003 19778545

[B25] Kusche-Vihrog K. Jeggle P. Oberleithner H. (2014). The role of ENaC in vascular endothelium. *Pflugers Arch.* 466 851–859. 10.1007/s00424-013-1356-3 24046153

[B26] Kusche-Vihrog K. Sobczak K. Bangel N. Wilhelmi M. Nechyporuk-Zloy V. Schwab A. (2008). Aldosterone and amiloride alter ENaC abundance in vascular endothelium. *Pflugers Arch.* 455 849–857. 10.1007/s00424-007-0341-0 17891415

[B27] Liang C. Wang Q. S. Yang X. Niu N. Hu Q. Q. Zhang B. L. (2017). Oxidized low-density lipoprotein stimulates epithelial sodium channels in endothelial cells of mouse thoracic aorta. *Br. J. Pharmacol.* 10.1111/bph.13853 [Epub ahead of print]. 28480509 PMC5866960

[B28] Liu H. B. Zhang J. Sun Y. Y. Li X. Y. Jiang S. Liu M. Y. (2015). Dietary salt regulates epithelial sodium channels in rat endothelial cells: adaptation of vasculature to salt. *Br. J. Pharmacol.* 172 5634–5646. 10.1111/bph.13185 25953733 PMC4667865

[B29] Lobbes M. B. Miserus R. J. Heeneman S. Passos V. L. Mutsaers P. H. Debernardi N. (2009). Atherosclerosis: contrast-enhanced MR imaging of vessel wall in rabbit model–comparison of gadofosveset and gadopentetate dimeglumine. *Radiology* 250 682–691. 10.1148/radiol.2503080875 19244042

[B30] Mammoto A. Mammoto T. Kanapathipillai M. Yung C. W. Jiang E. Jiang A. (2013). Control of lung vascular permeability and endotoxin-induced pulmonary oedema by changes in extracellular matrix mechanics. *Nat. Commun.* 4:1759. 10.1038/ncomms2774 23612300

[B31] Pedersen S. F. Thrysøe S. A. Paaske W. P. Thim T. Falk E. Ringgaard S. (2011). CMR assessment of endothelial damage and angiogenesis in porcine coronary arteries using gadofosveset. *J. Cardiovasc. Magn. Reson.* 13:10. 10.1186/1532-429X-13-10 21269470 PMC3036628

[B32] Pérez F. R. Venegas F. González M. Andrés S. Vallejos C. Riquelme G. (2009). Endothelial epithelial sodium channel inhibition activates endothelial nitric oxide synthase via phosphoinositide 3-Kinase/akt in small-diameter mesenteric arteries. *Hypertension* 53 1000–1007. 10.1161/HYPERTENSIONAHA.108.128520 19398659

[B33] Radeva M. Y. Waschke J. (2017). Mind the gap: mechanisms regulating the endothelial barrier. *Acta Physiol.* 222:e12860. 10.1111/apha.12860 28231640

[B34] Raitakari O. T. Celermajer D. S. (2000). Flow-mediated dilatation. *Br. J. Clin. Pharmacol.* 50 397–404. 10.1046/j.1365-2125.2000.00277.x 11069434 PMC2014404

[B35] Ren Q. Ren L. Ren C. Liu X. Dong C. Zhang X. (2015). Platelet endothelial cell adhesion molecule-1 (PECAM1) plays a critical role in the maintenance of human vascular endothelial barrier function. *Cell Biochem. Funct* 33 560–565. 10.1002/cbf.3155 26607202

[B36] Schneider J. C. El Kebir D. Chéreau C. Mercier J. C. Dall’Ava-Santucci J. Dinh-Xuan A. T. (2002). Involvement of Na^+^/Ca^2+^ exchanger in endothelial NO production and endothelium-dependent relaxation. *Am. J. Physiol. Heart Circ. Physiol.* 283 H837–H844. 12124234 10.1152/ajpheart.00789.2001

[B37] Tarjus A. Maase M. Jeggle P. Martinez-Martinez E. Fassot C. Loufrani L. (2017). The endothelial αENaC contributes to vascular endothelial function *in vivo*. *PLoS One* 12:e0185319. 10.1371/journal.pone.0185319 28950003 PMC5614594

[B38] Wang S. Meng F. Mohan S. Champaneri B. Gu Y. (2009). Functional ENaC channels expressed in endothelial cells: a new candidate for mediating shear force. *Microcirculation* 16 276–287. 10.1080/10739680802653150 19225981

[B39] Wang Z. R. Liu H. B. Sun Y. Y. Hu Q. Q. Li Y. X. Zheng W. W. (2017). Dietary salt blunts vasodilation by stimulating epithelial sodium channels in endothelial cells from salt-sensitive Dahl rats. *Br. J. Pharmacol.* 10.1111/bph.13817 [Epub ahead of print]. 28409833 PMC5866993

[B40] Warnock D. G. Kusche-Vihrog K. Tarjus A. Sheng S. Oberleithner H. Kleyman T. R. (2014). Blood pressure and amiloride-sensitive sodium channels in vascular and renal cells. *Nat. Rev. Nephrol.* 10 146–157. 10.1038/nrneph.2013.275 24419567 PMC4137491

[B41] Wiesinger A. Peters W. Chappell D. Kentrup D. Reuter S. Pavenstädt H. (2013). Nanomechanics of the endothelial glycocalyx in experimental sepsis. *PLoS One* 8:e80905. 10.1371/journal.pone.0080905 24278345 PMC3835794

