# Supplementary material for: The Deletion of Endothelial Sodium Channel α (αENaC) Impairs Endothelium-Dependent Vasodilation and Endothelial Barrier Integrity in Endotoxemia in Vivo
Source: Front Pharmacol. 2018 Apr 10;9:178. doi: 10.3389/fphar.2018.00178 (PMC5902527; doi:10.3389/fphar.2018.00178)
Supplement: Supplementary file 1 [file Data_Sheet_1.pdf]

Supplementary Material

The deletion of endothelial sodium channel α (αENaC) impairs endothelium-dependent vasodilation and endothelial barrier integrity in endotoxemia *in vivo*

Magdalena Sternak[1], Anna Bar[1,2], Mateusz G. Adamski[1], Tasnim Mohaissen[1,3], Brygida Marczyk[1], Anna Kieronska[1,2], Marta Stojak[1], Kamil Kus[1], Antoine Tarjus[4], Frederic Jaisser[4,5], Stefan Chlopicki[1,2]*

*** Correspondence:** Stefan Chlopicki, stefan.chlopicki@jcet.eu

1 Supplementary Data

Additional methodology

Short-term occlusion of femoral artery by the home-made vessel occluder

Vessel occlusion was performed using home-made vessel occluder, (composed of several drinking straws with total length about 70 cm), fishing line (about 160 cm) and syringe (10 ml). Fishing line was placed inside the connected straws creating small loop at the end of the tube (covered by protective, plastic cap to avoid crushing of the straw during vessel occlusion) and was connected with plunger of syringe (Fig. S1A). Vessel occluder was placed in measurement bed while loop was put on mice hind limb (Fig. S1B). At the beginning of the experiment, 3D image of femoral artery was obtained before vessel occlusion and then mice were pulled out from MRI scanner in order to provide proper loop positioning. Vessel occlusion was induced by pulling of the syringe plunger resulting in the loop closing. In the next step, positioning measurements were repeated and after 5 minutes of vessel occlusion the clamp was released by pushing the syringe plunger. 3D image of the femoral artery after vessel occlusion was obtained immediately after loop release.

To assess the percentage of flow reduction with the occluder the skin of the hind limb covering the artery was removed to gain access to the vessel area and effect of the vessel occlusion was observed using microscope. At complete flow reduction by vessel occluder, position of the syringe plunger was marked on syringe scale, what was helpful in performing repeatable vessels occlusions and *in vivo* measurements.

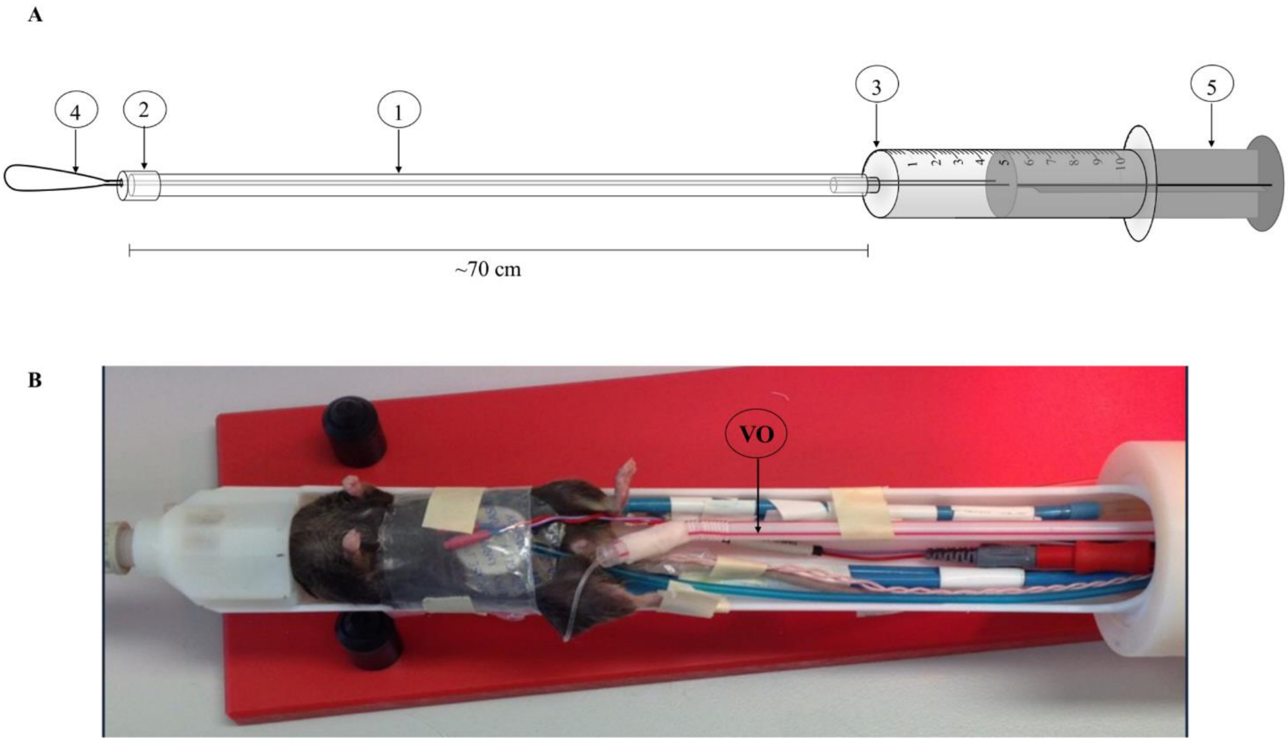

Fig.S1 Methodology of MRI-based flow-mediated dilatation measurements. **A**: Scheme of the vessel occluder. Home-made vessel occluder was composed of several connected drinking straws (1) covered by protective cap at the one end (2), syringe (3) and fishing line (4) combined with syringe plunger (5). **B**: C57BL/6 mouse in the measurement bed. Vessel occluder (VO) is placed in measurement bed, while loop of fishing line is put on mice hind limb.

2 Supplementary Figures and Tables

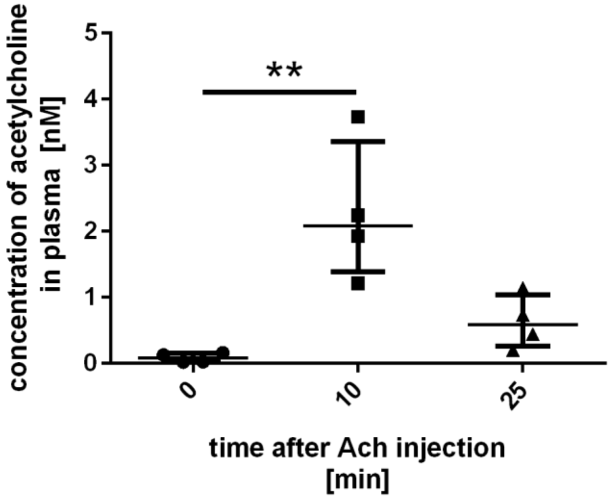

Fig.S2 Plasma concentration of acetylcholine (Ach) before, 10 and 25 minutes after Ach injection intraperitoneally, for measurement od endothelial-dependent vasodilation in control C57BL/6 mice. Statistics: Kruskal–Wallis test followed by Dunn’s post hoc test (normality was assessed using the Kolmogorov–Smirnov test). The results are presented as the median with interquartile range, for n=4 mice for each time point, $**p < 0.01$

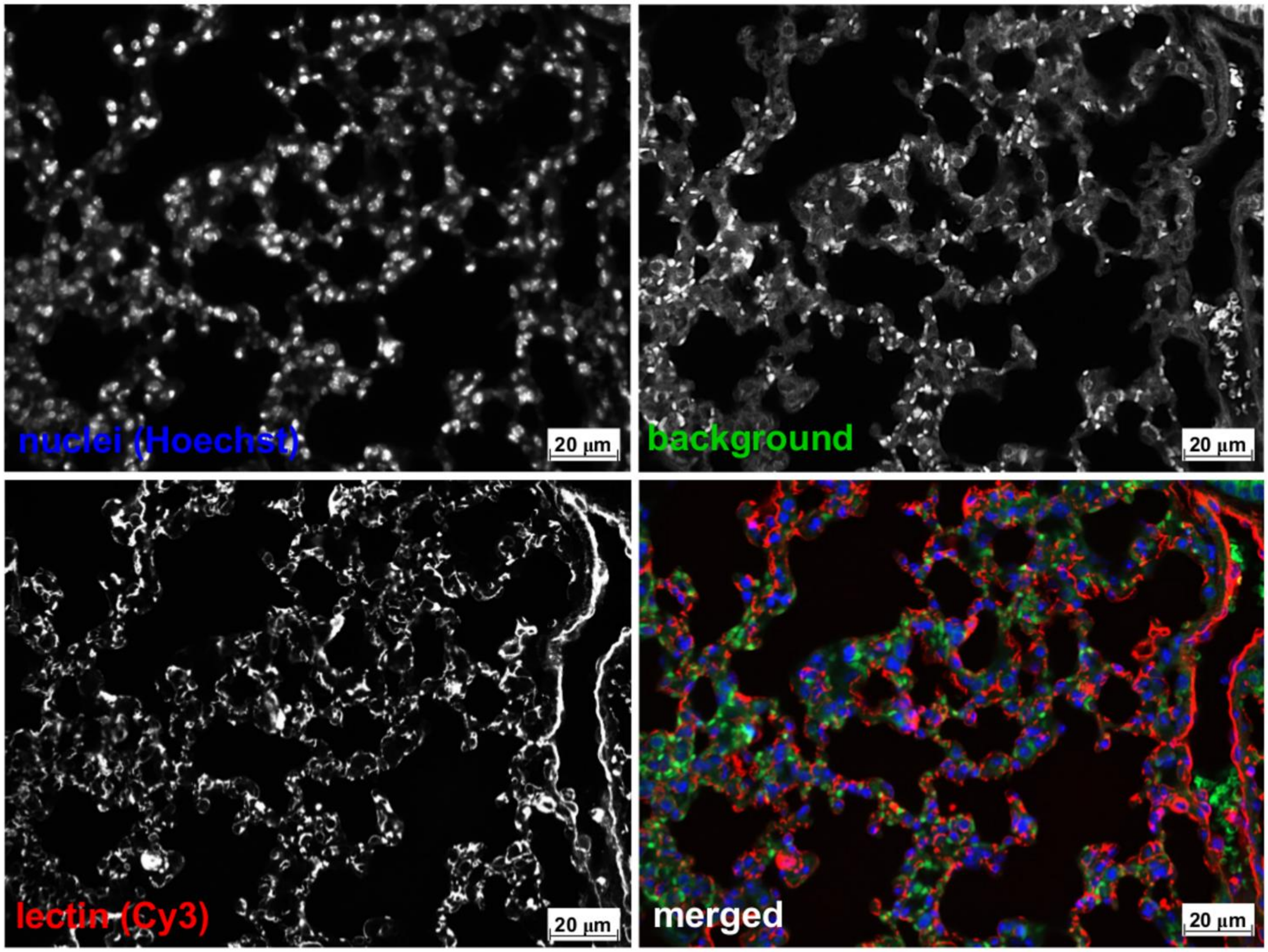

Fig.S3 Representative immunofluorescent images of lung microcirculation from control (saline) mice shown in three separate channels used for quantification of the relative lectin I immunopositive area in relation to the whole tissue area with the use of Columbus software. Immunopositive area was labelled by Cy3 (red channel), whole tissue area was shown as tissue autofluorescence (green channel, FITC filter without labelling). Nuclei were stained by Hoechst 33342 (blue channel).
